# Supplementary figures and images for: Identification of microRNAs involved in NOD-dependent induction of pro-inflammatory genes in pulmonary endothelial cells
Source: PLoS One. 2020 Apr 30;15(4):e0228764. doi: 10.1371/journal.pone.0228764 (PMC7192443; doi:10.1371/journal.pone.0228764)

S1 Fig

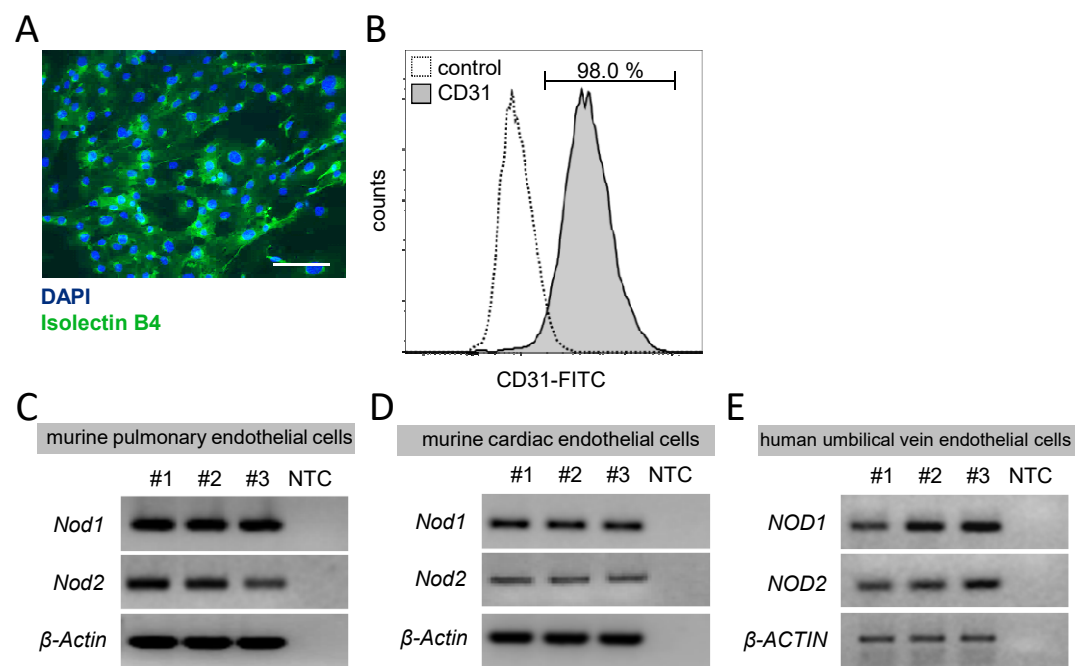

Supplement: S1 Fig — (A) Immunofluorescent staining of murine pulmonary endothelial cells with isolectin B4 and DAPI. A representative picture is shown. Scale bar = 50 μm. (B) Cell surface expression of CD31 on murine pulmonary endothelial cells analyzed by flow cytometry. Filled graphs represent CD31 expression, open graphs represent unstained controls. A representative experiment is shown. Nod1 and Nod2 mRNA expression was analyzed by real-time PCR in (C) murine pulmonary endothelial cells, in (D) murine cardiac endothelial cells and NOD1 and NOD2 mRNA in (E) human umbilical vein endothelial cells and loaded onto agarose gels. β-Actin/β-ACTIN is shown as loading control. Representative pictures are shown. NTC = no template control. (PDF) [file pone.0228764.s001.pdf]

S2 Fig

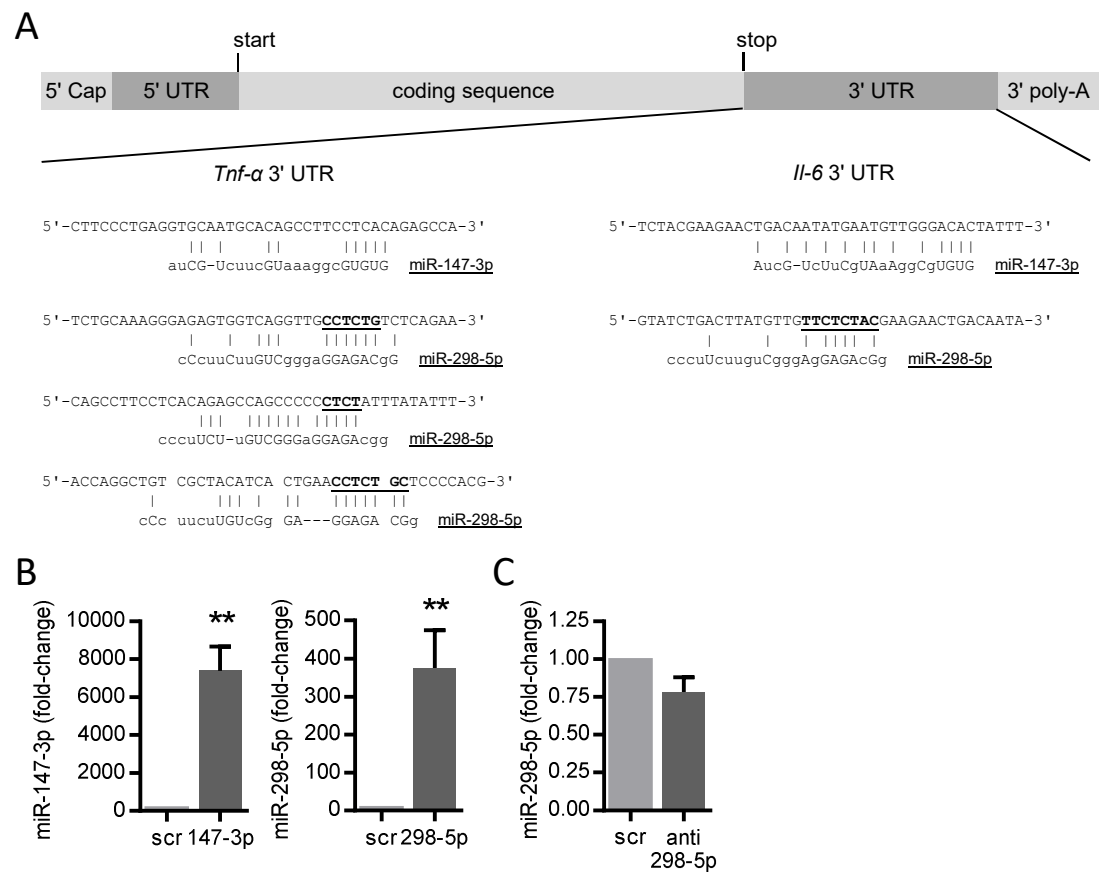

Supplement: S2 Fig — (A) Predicted binding sites for miR-147-3p and miR-298-5p in the 3' untranslated region (UTR) of the Tnf-α and Il-6 mRNA sequence. Seed sequences for miR-298-5p, which were deleted for luciferase reporter assays, are underlined and in bold. (B) miR-147-3p and miR-298-5p levels 24 hours after transfection of pulmonary endothelial cells with mimics for miR-147-3p or miR-298-5p and scrambled (scr) control (25 pmol each) was analyzed by real-time PCR. **p<0.01 vs. scr, n = 3–4. (C) miR-298-5p levels 24 hours after transfection of pulmonary endothelial cells with anti-miR-298-5p and scrambled (scr) control (25 pmol each) was analyzed by real-time PCR, n = 3. (PDF) [file pone.0228764.s002.pdf]

S3 Fig

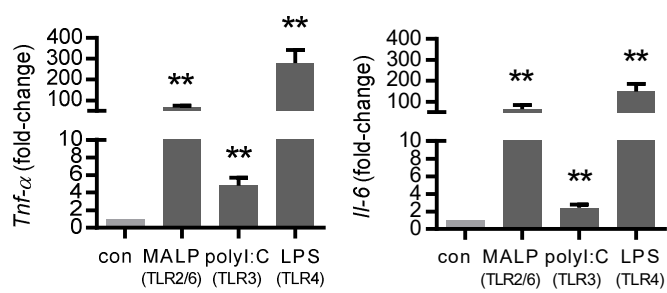

Supplement: S3 Fig — Cells were stimulated with the TLR2/6 agonist MALP-2 (1 μg/mL), with the TLR3 agonist polyI:C (1 μg/mL) or with the TLR4 agonist LPS (0.1 μg/mL) and Tnf-α and Il-6 mRNA expression was analyzed after 6 hours by real-time PCR. **P<0.01 vs. unstimulated control (con), n = 5–6. (PDF) [file pone.0228764.s003.pdf]
