# Supplementary material for: Identification of microRNAs involved in NOD-dependent induction of pro-inflammatory genes in pulmonary endothelial cells
Source: PLoS One. 2020 Apr 30;15(4):e0228764. doi: 10.1371/journal.pone.0228764 (PMC7192443; doi:10.1371/journal.pone.0228764)
Supplement: Attachment — Submitted filename: Response to Reviewers.docx [file pone.0228764.s004.docx]

**Response to Reviewers**

We highly appreciate the valued suggestions and criticism of the reviewer and the editor in order to strengthen the impact of our manuscript. Please find below a detailed response to all comments. Please note that we have added some replicates to single experiments in the course of the revision which leads to minor changes in bar graphs and significant values.

**Reviewer #1:**

Reviewer: The authors state that the expression level of miR-147 and miR-298 is low (CT=33), therefore, they only use overexpression experiments to demonstrate the role of these miRNA in the regulation of IL6 and TNF. However, the authors also claim that the downregulation of miR-147 and miR-298 participate to the upregulation of IL6 and TNF. Therefore, the authors should perform loss of function experiments to demonstrate that the downregulation of miR-147 and/or miR-298 participate to the control of TNF/IL6 expression (mRNA and protein). Alternatively, the authors could also try to perform experiment in which they overexpressed miR-298 in combination with target blocking site(s) oligos.

Authors: We used different methods, i.e. TaqMan low-density array, transfection of mimics and luciferase reporter assays to identify NOD-dependently expressed miRNAs to investigate for *Tnf-α* and *Il-6* mRNA regulation in murine pulmonary endothelial cells. Finally, we could confirm miR-298-5p from a couple of initially identified miRNAs by all methods and a direct physical interaction in case of *Il-6*. We have repeatedly stated this issue in the manuscript.

Following the remarks of the reviewer, we consequently focused on miR-298-5p and performed the suggested loss of function experiments using antagomirs for miR-298-5p and were able to further confirm our findings (result section on page 16/17 and novel Figure 4E and 4F and supplemental Figure S2C).

Reviewer: The authors should clearly state that the mechanism by which miR-147 participates to the control of IL6/TNF remains enigmatic

Authors: We explicitly stated now that we could not finally elucidate if miR-147-3p is involved in *Tnf-α* and *Il-6* regulation in pulmonary endothelial cells in this context (result section on page 16 and discussion section on page 20).

Reviewer: The authors should remove "In silico analysis" from the abstract as no in silico analysis were performed. At least for the reviewer, looking at putative binding sites in the 3'UTR of two transcripts do not account for in silico analysis.

Authors: We have deleted the term "In silico analysis" from the abstract, method and result sections.

Reviewer: In Figure 2 downregulated miRNA are in red while up-regulated miRNAs are in blue. It is confusing as in general up-regulated genes/miRNA are represented in red while downregulated genes are represented in blue.

Authors: We have changed the colors in the heatmap in Figure 2A according to the reviewers´ suggestion.

Reviewer: In the text the authors should clearly state that miR-147 is mmu-miR-147 which correspond to hsa-miR-147b. It is important, as the existence of hsa-miR-147a (miR-147) remains debate.

Authors: We have added this information to the result section on page 17.

Reviewer: The authors should acknowledge previous work on mmu-miR-147/hsa-miR-147b and the inflammatory responses.

Authors: Since we investigated both NOD-dependently down-regulated candidates from the initial array – miR-147 and miR-298 – we added additional previous work on inflammatory responses for both miRNAs (ref# 30-32) to the discussion section on page 20.

**Editor:**

Editor: You should clearly respond to the three major concerns raised by the Reviewer. In particular, the first major point about miR-147 and/or miR-298 function(s) appears essential and need additional experiments.

Authors: We addressed all concerns raised by the reviewer including additional loss of function experiments on miRNA function, please see above.

Editor: Moreover, I have also personally raised one additional point: while the authors claim that all data are available, they did not provide the full dataset of miRNA TLDA assay in a specific supplemental table (or a link to a public database).

Authors: We now provided the full dataset in the GEO public database (GSE145798) at NCBI and included this information in the method section on page 10 and in the result section on page 13.

At the moment, results are deposited as ‘private data’. In case of acceptance, we will set the status to ‘public data’. The reviewer may use: https://www.ncbi.nlm.nih.gov/geo/query/acc.cgi?acc=GSE145798 and token: atofyckibhmnnwt to view the full dataset.

**Journal Requirements:**

Please amend your current ethics statement to confirm that your named institutional review board or ethics committee specifically approved this study.

Authors: We have amended our ethics statement on page 6 in the method section as requested.
