# Supplementary material for: Identification of microRNAs involved in NOD-dependent induction of pro-inflammatory genes in pulmonary endothelial cells
Source: PLoS One. 2020 Apr 30;15(4):e0228764. doi: 10.1371/journal.pone.0228764 (PMC7192443; doi:10.1371/journal.pone.0228764)
Supplement: Attachment — Submitted filename: Response to Reviewers (R2).docx [file pone.0228764.s005.docx]

**Response to Reviewers**

**Reviewer #1:**

Reviewer: The authors should replace the term antagomiR by anti-miRNA, as antagomiR refer to a specific class of miR inhibitors (coupled to cholesterol allowing miRNA delivery without adding transfection reagent).

In addition, the authors should include the manufacturer of their anti-miRNA.

Authors: We have replaced the term antagomir by anti-miRNA throughout the manuscript and included the manufacturer (Dharmacon as for mimics) on page 9 of the material and method section.
